# Pituitary hemorrhage in pituitary adenomas treated with gamma knife radiosurgery: incidence, risk factors and prognosis

**DOI:** 10.7150/jca.52349

**Published:** 2021-01-01

**Authors:** Junyi Fu, Yanli Li, Lisha Wu, Xin Yang, Tingting Quan, Xi Li, Jiamin Zeng, Yinhui Deng, Jinxiu Yu

**Affiliations:** 1 Department of Neurology, The Second Affiliated Hospital of Guangzhou Medical University, Guangzhou, Guangdong, China. 510260.; 2 Department of Endocrinology, The Second Affiliated Hospital of Guangzhou Medical University, Guangzhou, Guangdong, China. 510260.; 3 Department of Medical Oncology, Sun Yat-sen Memorial Hospital, Sun Yat-sen University, Guangzhou, China. 510120.; 4 Department of Thoracic Surgery, The Second Affiliated Hospital of Guangzhou Medical University, Guangzhou, Guangdong, China. 510260.; 5 Department of Radiology, Sun Yat-sen University Cancer Center, State Key Laboratory of Oncology in South China, Collaborative Innovation Center for Cancer Medicine, Guangzhou, Guangdong, China, 510060.; 6 Department of Radiology, The Second Affiliated Hospital of Guangzhou Medical University, Guangzhou, Guangdong, China. 510260.; 7 Department of Pathology, The Second Affiliated Hospital of Guangzhou Medical University, Guangzhou, Guangdong, China. 510260.; 8 Department of Radiotherapy, The Second Affiliated Hospital of Guangzhou Medical University, Guangzhou, Guangdong, China. 510260.

**Keywords:** gamma knife, radiosurgery, pituitary apoplexy, pituitary hemorrhage, pituitary adenoma

## Abstract

**Objective**: The aims of this study were to investigate the incidence, risk factors and prognosis of pituitary hemorrhage in pituitary adenomas treated with gamma knife radiosurgery (GKRS).

**Methods and materials:** Between December 1993 and December 2016, 751 consecutive pituitary adenoma patients treated with GKRS were retrospectively reviewed in a single center. There were 271 male (36.1%) and 480 female (63.9%) patients with a median age of 38.5 (range, 7.2-84.0) years. The number of nonfunctioning pituitary adenomas (NFPAs) and functioning pituitary adenomas were 369 (49.1%) and 382 (50.9%) respectively. The median follow-up time was 61.1 (range, 12.1-304.4) months.

**Results:** In this study, 88 patients (11.7%) were diagnosed with pituitary hemorrhage before GKRS, 55 patients (7.3%) developed new or worsened pituitary hemorrhage after GKRS (excluding 3 patients with new or worsened pituitary hemorrhage due to tumor regrowth). The median time to new or worsened pituitary hemorrhage after GKRS was 18.9 (range 3.1-70.7) months. Overall, 128 patients (17.0%) were diagnosed with pituitary hemorrhage in the entire series. After adjustment with logistic regression, nonfunctioning pituitary adenomas (NFPAs) (odds ratio [OR]=2.121, 95% confidence interval [CI]=1.195-3.763, *p*=0.010) and suprasellar extension (OR=2.470, 95% CI=1.361-4.482, *p*=0.003) were associated with pituitary hemorrhage before GKRS. NFPA (OR=3.271, 95% CI=1.278-8.373, *p*=0.013) was associated with new or worsened pituitary hemorrhage after GKRS. Five patients received surgical resection for new or worsened pituitary hemorrhage were considered as GKRS treatment failure. Two patients with new hypopituitarism were considered to be owed to new or worsened pituitary hemorrhage after GKRS.

**Conclusions:** New or worsened pituitary hemorrhage after GKRS was not an uncommon phenomenon. NFPA was an independent risk factor of new or worsened pituitary hemorrhage after GKRS. New or worsened pituitary hemorrhage after GKRS could lead to GKRS treatment failure. GKRS might be a precipitating factor of pituitary hemorrhage.

## Introduction

Pituitary adenomas are one of the most frequent intracranial primary tumors, representing about 16.7% of all brain tumors 1. These adenomas can be classified as nonfunctioning pituitary adenomas (NFPAs) and functioning pituitary adenomas (FPAs) according to the evidence of secreting biologically active hormones.

Clinical pituitary apoplexy is a rare disorder with an acute clinical syndrome characterized by severe headaches, sudden visual impairment and ophthalmoplegia due to hemorrhage or infarction of pituitary adenomas and related with significant morbidity. It may present more common in NFPAs than FPAs 2. Some epidemiological studies reported its incidence was 0.17 episodes per 100 000 per year 3 and 6.2 cases per 100 000 inhabitants 4. Almost 0.6%-10.0% of pituitary adenoma patients experience apoplexy 2, 5-7. Comparing to acute apoplexy, subclinical apoplexy based on radiological findings or autopsy which is asymptomatic, is more common, with up to 25% of pituitary adenomas 8-13. Pituitary hemorrhage is the main form of clinical or subclinical pituitary apoplexy, with up to 28.1% in pituitary adenomas 14.

Predisposing and precipitating factors of pituitary hemorrhage including arterial hypertension, intracranial pressure, major surgery, closed head trauma, dynamic tests, angiography, dopamine agonists (DAs) and anticoagulant therapy had been widely reported 9. Radiation-induced pituitary hemorrhage had been reported previously. But only a small number of cases were reported thus far 15-19. In our previous study 20, the early pattern of treatment failure in initial Gamma Knife radiosurgery (GKRS) for NFPAs was pituitary apoplexy. Seven patients (8.6%) developed new pituitary hemorrhage after GKRS. Tumor volume (≥10 cm^3^) was an independent risk factor. GKRS has been the most commonly used radiation technique and essential part in the treatment of pituitary adenomas, with advantages of better dose conformity, a highly precise and focused delivery of radiation in a single session. However, little is known about the incidence, risk factors and prognosis of pituitary hemorrhage in pituitary adenoma patients treated with GKRS so far.

There are more than 26 years' experience using GKRS (Elekta, Stockholm, Sweden) for pituitary adenomas at the Second Affiliated Hospital of Guangzhou Medical University since 1993. In order to report the incidence, risk factors and prognosis of pituitary hemorrhage in pituitary adenomas treated with GKRS, we performed a large single-center retrospective study.

## Methods

### Patient population

Between December 1993 and December 2016, the medical records of 2557 patients who underwent GKRS for the treatment for pituitary adenomas were retrospectively reviewed at the Second Affiliated Hospital of Guangzhou Medical University. 1806 cases of patients who were absence of clinical data and follow-up information in our hospital were excluded. Finally, 751 patients with available clinical data and at least one follow-up were included in this study. This retrospective study was approved by the institutional committee of the Second Affiliated Hospital of Guangzhou Medical University.

### Definition of pituitary hemorrhage in pituitary adenomas

**Figure 1:**
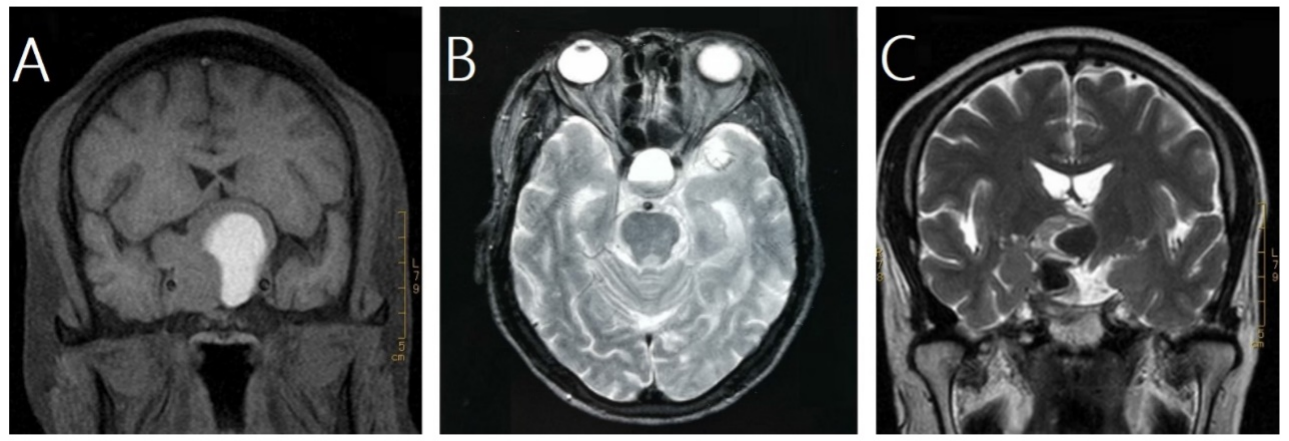
** Magnetic resonance images of three cases of pituitary hemorrhage. A**, a 43-year-old male with nonfunctioning pituitary adenoma (NFPA) presenting as a high intensity area on a coronal T1-weighted image. **B**, a 69-year-old male with NFPA showing the sign of a fluid-fluid level on an axial T2-weighted image. **C**, a 47-year-old male with NFPA presenting as a very low intensity area on a coronal T2-weighted image.

In this study, pituitary hemorrhage was diagnosed according to magnetic resonance imaging (MRI), computed tomography (CT), surgical findings or pathological findings. All of patients had MRI of sellar before and after GKRS. Images were reviewed by an experienced radiologist to confirm pituitary hemorrhage. The diagnosis of pituitary hemorrhage in MRI findings was mainly based on the following signs 21-23: (1) hyper intense signal on T1-weighted images (acute-subacute phase, Fig. 1A), (2) a fluid-fluid level in the mass (chronic phase, Fig. 1B), (3) very low intensity of hemosiderin on T2-weighted images (chronic phase, Fig. 1C).

### Clinical and radiological evaluations

Patients were required to take routine clinical and radiological follow-up evaluations every 6 months for the first 3 years and thereafter yearly. Clinical pituitary apoplexy was diagnosed based on episode of acute severe headaches, sudden visual disturbance or opthalmoplegia as well as presence of a pituitary adenoma. Subclinical pituitary apoplexy was defined as an intratumoral hemorrhage based on MRI, CT, surgical or pathological findings, with no clinical symptoms of acute pituitary adenoma apoplexy. Tumor dimensions were obtained manually from MRI. The dimensional indices of the tumors were measured and recorded in three orthogonal planes: transverse (TR), anteroposterior (AP), and craniocaudal (CC). Tumor volumes were estimated using the following formula: V = (π × [TR × AP × CC])/6 24. Parasellar invasion was defined as Knosp grade 3 or 4. Suprasellar extension was defined as the presence of a tumor close to optic nerve and chiasm (< 2mm). Predisposing factors of pituitary hemorrhage, including hypertension, diabetes mellitus, DAs, anticoagulants, and angiography, were reviewed in the medical records for all patients.

Hypopituitarism was defined asymptomatic anterior pituitary deficits with laboratory documentation. New hypopituitarism was defined by requiring hormone replacement or a new deficiency in any one of the hormonal axes after GKRS. Hypothyroidism was based on low free thyroxine (normal range, 12.00-22.00pmol/L). Hypocortisolism was defined if morning cortisol level (08:00) was <100 nmol/L. If morning cortisol level was >400 nmol/L, hypocortisolism was excluded. Hypogonadism in males was defined by low serum testosterone levels (<12 nmol/L) without elevated luteinizing hormone (LH) and follicle stimulating hormone (FSH) levels. Hypogonadism in females was defined by amenorrhea with low serum estradiol and low gonadotrophins in premenopausal women, and the absence of high gonadotrophins (LH and FSH) in menopausal women.

### Gamma knife radiosurgery technique

All of patients underwent GKRS in our hospital. The procedure was performed using model B Leksell Gamma Knife Unit before April 2014 and Perfexion Unit (Elekta Instrument, Inc. Stockholm, Sweden) thereafter. Following stereotactic Leksell frame placement, thin-slice stereotactic MRI with contrast was performed through the sellar. Subsequently, GKRS treatment planning was made in consultation with a medical physicist, radiation oncologist and neurosurgeon. Dose selection for GKRS was mainly dependent on the tumor type, tumor volume, distance to the optic nerve and chiasm. The median tumor margin dose was 14.4 Gy (range, 8.0-30.0 Gy) at a median prescription isodose 40% (range, 18.8-80.0%). The median maximum dose was 33.3 Gy (range, 14-90 Gy).

### Statistical analysis

The normal distribution of continuous variables was checked by Kolmogorov-Smirnov test. The median and interquartile ranges were used to describe variables not normally distributed. When continuous variables were not normally distributed, Wilcoxon rank sum test was used. Chi-square test and Fisher exact test were used for statistical analysis of categorical variables. Logistic regression was performed using variables that were significantly different between the hemorrhage and nonhemorrhage group on univariate analysis (*P* < 0.05), or nearing statistical significance (defined as *P* < 0.1). Probability values <0.05 were defined as statistically significant. For statistical analysis, IBM's SPSS (version 21.0) was used.

## Results

### Patients' characteristics

Seven hundred and fifty-one patients who had undergone GKRS between December 1993 and December 2016 were selected from the single-center. The patient population consisted of 271 men (36.1%) and 480 women (63.9%) patients with a median age of 38.5 (range, 7.2-84.0) years. The number of NFPAs and FPAs were 369 (49.1%) and 382 (50.9%) respectively. The median follow-up was 61.1 (range, 12.1-304.4) months. The median tumor volume at diagnosis was 2.4 (range, 0.02-58.70) cm^3^. There were 356 patients (47.4%) with suprasellar extension and 197 patients (26.2%) with parasellar invasion respectively.

### Incidence of pituitary hemorrhage

According to the criteria of diagnosis, 128 patients (17.0%) presented with pituitary hemorrhage. Of the 128 patients, 20 patients were clinical pituitary apoplexy. All of the patients treated with GKRS in our center. Before GKRS, there were 88 patients (11.7%) diagnosed with pituitary hemorrhage. Of the 88 patients, 14 patients were clinical pituitary apoplexy. After GKRS, there were 58 patients (7.7%) developed new or worsened pituitary hemorrhage. Of the 58 patients, 7 patients were clinical pituitary apoplexy, 18 patients also developed pituitary hemorrhage before GKRS. There was no difference between the proportion of clinical pituitary apoplexy in patients with pituitary hemorrhage before and after GKRS (*p*=0.518). The median time of new or worsened pituitary hemorrhage after GKRS was 20.5 (range, 3.1-199.2) months. Of the 58 patients, 3 patients with new or worsened pituitary hemorrhage were due to tumor regrowth at 146.5, 166.8 and 199.2 months respectively. After excluding the 3 patients, 55 patients (7.3%) were left, the median time of new or worsened pituitary hemorrhage was 18.9 (range 3.1-70.7) months.

### Risk factors associated with pituitary hemorrhage

A number of clinical characteristics were analyzed according to different groups (Table 1). In univariate analysis, age, NFPAs, tumor volumes (TV) at diagnosis, suprasellar extension and hypertension were associated with hemorrhage before GKRS. Male, age, NFPAs, TV at GKRS, surgery, margin dose, suprasellar extension and DAs were associated with new or worsened hemorrhage after GKRS. Male, age, NFPAs, TV at diagnosis and suprasellar extension were associated with pituitary hemorrhage (total). After adjustment with logistic regression, NFPAs (odds ratio [OR]=2.121, 95% confidence interval [CI]=1.195-3.763, *p*=0.010) and suprasellar extension (OR=2.470, 95% CI=1.361-4.482, *p*=0.003) were associated with hemorrhage before GKRS. NFPAs (OR=3.271, 95% CI=1.278-8.373, *p*=0.013) was independent risk factor of new or worsened hemorrhage after GKRS. Totally, for all of the 128 patients with pituitary hemorrhage, NFPAs (OR=2.528, 95% CI=1.538-4.155, *p*=0.000) and suprasellar extension (OR=2.589, 95% CI=1.552-4.317, *p*=0.000) were independent risk factors.

### Pituitary hemorrhage and tumor control

In the study, there were 28 patients developed tumor regrowth after GKRS. Pituitary hemorrhage before GKRS was not associated with tumor regrowth by log-rank test (*p*=0.618). New or worsened pituitary hemorrhage after GKRS was not associated with tumor regrowth by Chi-square test (*p*=0.092). The median time to tumor shrinkage after GKRS was 14.9 (range, 2.6-216.1) months. Of the 58 patients with new or worsened pituitary hemorrhage, 5 patients receiving surgical resection for clinical pituitary apoplexy were defined as GKRS treatment failure. Four patients developed tumor regrowth, including 1 patient receiving surgery for clinical pituitary apoplexy due to tumor regrowth. Six patients who were confirmed as tumor enlargement due to pituitary hemorrhage after GKRS were under observation and still considered as tumor control. Four patients remained stable. Thirty-nine tumors were shrinkage. New or worsened pituitary hemorrhage could result in treatment failure after GKRS.

### Pituitary hemorrhage and pituitary function

Of the 88 patients with pituitary hemorrhage before GKRS, only 65 patients (73.9%) had endocrine evaluations before GKRS in our hospital. Of the 65 patients, 33 patients (50.8%) presented with hypopituitarism at diagnosis. The number of hypothyroidism, hypocortisolism and hypogonadism were 11 (16.9%), 10 (15.4%) and 26 (40.0%) respectively. It was difficult to investigate the relationship between hypopituitarism and pituitary hemorrhage before GKRS. Of the 58 patients with new or worsened pituitary hemorrhage, only 49 patients (84.5%) had endocrine evaluations after GKRS in our hospital. Of the 49 patients, 30 patients (61.2%) presented with hypopituitarism. The number of hypothyroidism, hypocortisolism and hypogonadism were 18 (36.7%), 12 (24.5%) and 28 (57.1%), respectively. Thirteen patients (26.5%) developed new hypopituitarism in the group of new or worsened pituitary hemorrhage after GKRS. GKRS, surgery, compression of pituitary gland by tumor and pituitary apoplexy could develop new hypopituitarism after GKRS. Of the 13 patients, 2 patients with new hypopituitarism were considered to be caused by new or worsened clinical pituitary apoplexy after GKRS.

## Discussion

This was the largest study describing the incidence, risk factors and prognosis of pituitary hemorrhage in pituitary adenomas treated with GKRS in a single-center. Clinical pituitary apoplexy is a rare event with a prevalence of 0.6%-10% in previous studies 2, 5-7. In contrast, pituitary hemorrhage including clinical and subclinical pituitary apoplexy, is not an uncommon phenomenon with an incidence of 14%-28% 5, 14, 25. Symon et al. 26 reported the incidence of pituitary hemorrhage was 18.1% based on 320 cases of postsurgical specimens. However, this was a cohort with a high proportion of giant or large recurrent pituitary adenomas. Yousem et al. 27 reported a prevalence of 26% of pituitary hemorrhage based on MRI or surgical findings in 68 pituitary adenomas treated medically or surgically, or with radiotherapy. In the study of Liu et al. 14, the incidence of pituitary hemorrhage was 28.1%. In this study, 89.2% of patients were macroadenomas. In the current study, our data showed the incidence of pituitary hemorrhage was 17.0% in patients treated with GKRS totally, which was similar with previous studies 5, 14, 25. Eighty-eight patients (11.7%) presented with pituitary hemorrhage before GKRS, which were mainly spontaneous hemorrhage. Fifty-eight patients (7.7%) developed new or worsened pituitary hemorrhage after GKRS. After excluding the 3 patients with new or worsened pituitary hemorrhage due to tumor regrowth, 55 patients (7.3%) were left. This was the first study reporting the incidence of new or worsened pituitary hemorrhage after GKRS. The median time of the 55 patients developed new or worsened pituitary hemorrhage was 18.9 (range 3.1-70.7) months. Pituitary adenomas could present with spontaneous hemorrhage. As the high incidence of new or worsened pituitary hemorrhage after GKRS, GKRS might play an important role in the pathogenesis of pituitary hemorrhage. GKRS might be a precipitating factor of pituitary hemorrhage.

Predisposing and precipitating factors of pituitary hemorrhage including arterial hypertension, intracranial pressure, major surgery, closed head trauma, dynamic tests, angiography, DAs, radiotherapy and anticoagulant therapy have been widely reported 9, 15. However, only a small number of cases with pituitary hemorrhage associated with radiation were reported. Literature reviews on the precipitating factor of radiation for pituitary hemorrhage were showed in Table 3. The incidence, risk factors and prognosis of pituitary hemorrhage in pituitary adenoma patients treated with GKRS have not been reported thus far. In 1977, Weisberg et al 15 reported 14 of 300 patients developed clinical pituitary apoplexy. Among of them, 8 patients with pituitary apoplexy were associated with radiotherapy. Wakai et al. 10 investigated the occurrence of pituitary hemorrhage from 560 cases of pituitary adenomas treated with operation. Apoplectic episodes occurred shortly after the completion of postoperative radiotherapy in 3 cases. One patient occurred hemorrhage with no acute episode after radiation. Zhang et al. 28 investigated the clinical features, diagnosis, treatment and outcomes of subclinical pituitary apoplexy in 185 consecutive patients. Among of them, 5 patients received preoperative radiotherapy. Radiation might be potentially risky in these patients.

The pathophysiology of pituitary hemorrhage is not completely understood. Vascular flux reduction and intra or postoperative hypotension may be proposed mechanisms of pituitary hemorrhage after surgical procedures 29, 30. Pituitary hemorrhage after angiographic procedures may be related either to vasospasm or blood pressure fluctuations 31. The increased metabolic needs of the tumors induced by dynamic testing may cause pituitary hemorrhage 32, 33. Patients treated with anticoagulation therapy have an unavoidable risk of bleeding 34. Besides, pituitary hemorrhage can occur spontaneously without any precipitating factor. A rapidly expanding adenomas with poor vascularity that outgrow their blood supply or because ischemia (and thus infarction) occurs following compression of infundibular or superior hypophyseal vessels may result in pituitary hemorrhage 35, 36. Because of the infrequent occurrence, and lacking of case control study, a direct causal relationship between radiation and pituitary hemorrhage has not been confirmed. It is postulated that pituitary hemorrhage may occur following tumor infarction or necrosis after GKRS. Vascular changes after pituitary irradiation often result in chronic hypoperfusion of the pituitary gland and have been associated with both pituitary infarction and hemorrhage 37. In addition, GKRS might damage the tumor neovascularity to produce hemorrhage 38. Interestingly, the median time of tumor shrinkage was 14.9 months in this study, which was a little shorter than that of new or worsened pituitary hemorrhage after GKRS. Because of the high incidence and interesting occurrent time, new or worsened pituitary hemorrhage after GKRS might not be a coincidence.

In the current study, pituitary hemorrhage before GKRS was significantly associated with NFPAs and suprasellar extension. New or worsened pituitary hemorrhage after GKRS was related with NFPAs. We also investigated the relationship between gender, age, tumor volume, surgery, parasellar invasion, hypertension, diabetes mellitus, DAs, anticoagulants, angiography and pituitary hemorrhage, but did not find any association. Pituitary apoplexy was observed more frequently in NFPA patients than functioning tumors 39. Moller-Goede et al. 39 analyzed incidence and risk factors of clinical pituitary apoplexy by reviewing 574 patients with pituitary adenoma. Forty-two patients (7.3%) were diagnosed as clinical pituitary apoplexy. The incidence of clinical pituitary apoplexy in nonfunctioning tumors and functioning tumor were 16.5% and 5.0% respectively. Male and nonfunctioning tumor type were found to be independent risk factors for clinical pituitary apoplexy (*p*<0.001). Suprasellar extension was associated with pituitary hemorrhage before GKRS. Perhaps, suprasellar extension can compress the infundibular or superior hypophyseal arteries 40, and be risky for pituitary hemorrhage.

In the current study, we did not find relationship between pituitary hemorrhage and tumor regrowth after GKRS. However, every coin has two sides. The course of new or worsened pituitary hemorrhage after GKRS was variable. New or worsened pituitary hemorrhage after GKRS could result in GKRS treatment failure or tumor shrinkage. On the one hand, acute headache, visual abnormalities, hypopituitarism might occur due to tumor enlargement following new or worsened pituitary hemorrhage after GKRS in some cases, and need surgical resection. Of the 58 patients with new or worsened pituitary hemorrhage after GKRS, 5 patients received surgical resection for clinical pituitary apoplexy were defined as GKRS treatment failure. On the other hand, pituitary adenomas could be destroyed by new or worsened pituitary hemorrhage after GKRS. Tumor shrinkage might be accelerated by hemorrhage after GKRS. Therefore, we postulated one of potential mechanisms of GKRS treatment for pituitary adenomas may via radiation-induced pituitary hemorrhage.

Although the current study is the largest describing the incidence, risk factors and prognosis of pituitary hemorrhage in pituitary adenomas treated with GKRS in a single-center, there were several limitations should be noticed. Firstly, it was a single-center retrospective study and there were information and selection biases. Because many patients came from long distance, there were only 741 patients undergoing follow-up evaluation in our hospital. Thus, many patients were lost to follow up. It could cause selection bias in this study. Secondly, the diagnosis of pituitary hemorrhage was mainly based on MRI. Only a small proportion of patients underwent surgery. Besides, some patients did not undergo regular follow-up MRI. Therefore, some micro-bleedings in pituitary adenomas might be missing. Thirdly, this was not a case-control study, the role and mechanism of GKRS on new or worsened pituitary hemorrhage after GKRS needed further study.

To conclude, we have demonstrated the incidence, risk factors and prognosis of pituitary hemorrhage in pituitary adenoma patients treated with GKRS. In this study, 11.7% of patients were diagnosed with pituitary hemorrhage before GKRS, 7.3% of patients developed new or worsened pituitary hemorrhage after GKRS (excluding 3 patients with new or worsened pituitary hemorrhage due to tumor regrowth). The median time to new or worsened pituitary hemorrhage after GKRS was 18.9 (range 3.1-70.7) months. Overall, 17.0% patients were diagnosed with pituitary hemorrhage in the entire series. NFPAs and suprasellar extension were associated with pituitary hemorrhage before GKRS. NFPA was an independent risk factor for new or worsened pituitary hemorrhage after GKRS. New or worsened pituitary hemorrhage after GKRS could result in GKRS treatment failure. GKRS might be a precipitating factor of pituitary hemorrhage.

## Figures and Tables

**Table 1:** Unadjusted comparison of clinical characteristics in 751 patients with pituitary adenomas

Characteristics	Hemorrhage before GKRS			New or worsened hemorrhage after GKRS			Pituitary hemorrhage (total)		
	Yes (n=88)	No (n=663)	*P* value	Yes (n=58)	No (n=693)	*P* value	Yes (n=128)	No (n=623)	*P* value
Gender (male)	38 (43.2%)	233 (35.1%)	0.140	33 (56.9%)	238 (34.3)	0.001^*^	60 (46.9%)	211 (33.9%)	0.005^*^
Age at GKRS, y	43.8 (33.7-55.8)	38.1 (29.1-48.8)	0.004^*^	48.1 (37.8-58.8)	38.0 (29.1-48.6)	0.000^*^	44.2 (35.8-56.6)	37.5 (29.0-48.1)	0.000^*^
NFPAs	66 (75%)	303 (45.7%)	0.000^*^	48 (82.8%)	321 (46.3%)	0.000^*^	98 (76.6%)	271 (43.5%)	0.000^*^
TV at diagnosis, ml	6.0 (2.1-11.0)	2.4 (0.5-7.2)	0.000^*^	NA	NA	NA	5.4 (2.2-11.0)	2.1 (0.4-7.1)	0.000^*^
TV at GKRS, ml	NA	NA	NA	4.0 (2.5-8.8)	1.5 (0.5-4.7)	0.000^*^	NA	NA	NA
Surgery	NA	NA	NA	24 (41.4%)	187 (27.0%)	0.019^*^	NA	NA	NA
Margin dose at GKRS, Gy	NA	NA	NA	12.8 (10.7-14.1)	15.0 (12.8-16.5)	0.000^*^	NA	NA	NA
Suprasellar extension	66 (75%)	290 (43.7%)	0.000^*^	46 (79.3%)	310 (44.7%)	0.000^*^	96 (75%)	260 (41.7%)	0.000^*^
Parasellar invasion	26 (29.5%)	171 (25.8%)	0.452	16 (27.6%)	181 (26.1%)	0.807	35 (27.3%)	162 (26.0%)	0.754
Hypertension	14 (15.9%)	58 (8.7%)	0.032^*^	8 (13.8%)	64 (9.2%)	0.257	18 (14.1%)	54 (8.7%)	0.059
Diabetes mellitus	6 (6.8%)	19 (2.9%)	0.104	3 (5.2%)	22 (3.2%)	0.664	7 (5.5%)	18 (2.9%)	0.226
DAs	16 (18.2%)	149 (22.5%)	0.361	6 (10.3%)	159 (22.9%)	0.026^*^	22 (17.2%)	143 (23.0%)	0.151
Anticoagulants	4 (4.5%)	11 (1.7%)	0.158	3 (5.2%)	12 (1.7%)	0.190	4 (3.1%)	11 (1.8%)	0.513
Angiography	2 (2.3%)	6 (1.0%)	0.239	1 (1.7%)	7 (1.0%)	0.476	2 (1.6%)	6 (1.0%)	0.897

Data are expressed as number, median and IQR, or percentage. Abbreviations: GKRS, gamma knife radiosurgery; IQR interquartile range; NFPA, nonfunctioning pituitary adenoma; TV, tumor volume; DA, dopamine agonist; NA, not available. *Statistically significant (*P* <0.05).

**Table 2:** Adjusted risk factors predicting pituitary hemorrhage in 751 patients with pituitary adenomas

Characteristics	Hemorrhage before GKRS (n=88)			New or worsened hemorrhage after GKRS (n=58)			Pituitary hemorrhage (total) (n=128)		
	OR	95% CI	*P* value	OR	95% CI	*P* value	OR	95% CI	*P* value
Gender (male)	NA	NA	NA	1.476	0.811-2.685	0.202	0.935	0.609-1.434	0.757
Age at GKRS	1.006	0.990-1.022	0.453	1.012	0.995-1.030	0.178	1.012	0.998-1.025	0.088
NFPAs	2.121	1.195-3.763	0.010^*^	3.271	1.278-8.373	0.013^*^	2.528	1.538-4.155	0.000^*^
TV at diagnosis	1.006	0.980-1.033	0.650	NA	NA	NA	1.002	0.978-1.026	0.884
TV at GKRS	NA	NA	NA	0.997	0.956-1.040	0.905	NA	NA	NA
Margin dose at GKRS	NA	NA	NA	0.910	0.796-1.041	0.170	NA	NA	NA
Surgery	NA	NA	NA	0.874	0.470-1.627	0.672	NA	NA	NA
Suprasellar extension	2.470	1.361-4.482	0.003^*^	1.959	0.872-4.401	0.103	2.589	1.552-4.317	0.000^*^
Hypertension	1.444	0.717-2.910	0.304	NA	NA	NA	1.111	0.586-2.106	0.746
DAs	NA	NA	NA	1.488	0.498-4.447	0.477	NA	NA	NA

Abbreviations: GKRS, gamma knife radiosurgery; NFPA, nonfunctioning pituitary adenoma; TV, tumor volume; DA, dopamine agonist; NA, not available; OR, odds ratio; CI, confidence interval. *Statistically significant (*P* <0.05).

**Table 3:** Literature review on the precipitating factor of radiation for pituitary hemorrhage

Studies/years	Patients, n	Number of pituitary hemorrhage	Number of pituitary hemorrhage associated with radiotherapy	Technique of radiotherapy	Precipitating factors for pituitary hemorrhage
Weisberg et al/1977 15	300	14 (pituitary apoplexy)	8 (pituitary apoplexy)	Radiocobalt; Linear electron accelerator with rotational technic	Radiotherapy
Wakai et al/1981 10	560	93 (pituitary hemorrhage)	3 (clinical pituitary apoplexy) (subclinical pituitary apoplexy)	NA	Postoperative radiotherapy, age Bromocriptine treatment
Semple et al/2005 19	62	62 (pituitary apoplexy)	(pituitary apoplexy)	NA	Radiotherapy, cardiac surgery, head trauma
Semple et al/2007 18	38	38 (pituitary apoplexy)	1 (pituitary apoplexy)	Gamma Knife radiosurgery	Gamma Knife irradiation, coronary artery surgery, other major surgery, pregnancy, anticoagulant therapy, coagulopathy secondary to liver failure
Zhang et al/2009 28	185	185 (subclinical pituitary apoplexy)	(subclinical pituitary apoplexy)	NA	Radiotherapy, hypertension, diabetes, closed head trauma, anticoagulant treatment

Abbreviations: NA, not available.
